# Simultaneous Determination of Six Active Components in Danggui Kushen Pills via Quantitative Analysis of Multicomponents by Single Marker

**DOI:** 10.1155/2019/9620571

**Published:** 2019-01-23

**Authors:** Huiwei Bao, Jihong Chi, Huailei Yang, Fangxin Liu, Kuo Fang, Yang Xu

**Affiliations:** ^1^College of Pharmacy, Changchun University of Chinese Medicine, Changchun, Jilin 130117, China; ^2^College of Pharmacy, Changchun Medical College, Changchun, Jilin 130000, China; ^3^Chinese Institute of Jilin Ginseng, Changchun, Jilin 130000, China

## Abstract

In this paper, a valid evaluation method for the quality control of Danggui Kushen pills (DKP) has been established based on quantitative analysis of multicomponents by single marker (QAMS). Gallic acid, matrine, oxymatrine, catechin, ferulic acid, and rutin were selected as the indexes for quality evaluation of DKP. The analysis was achieved on an Agilent ZORBAX SB-C18 column (250  mm × 4.6  mm, 5 *μ*m) via gradient elution. Gallic acid was used as internal standard to determine the relative correction factors (RCF) between gallic acid and other five constituents in DKP. The contents of those components were calculated at the same time. The accuracy of QAMS method was verified by comparing the contents of six components calculated by external standard (ES) method with those of the QAMS method. It turned out that there was no significant difference between the quantitative results of QAMS method and external standard method. The proposed QAMS method was proved to be accurate and feasible according to methodological experiments, which provided an accurate, efficient, and economical approach for quality evaluation of DKP.

## 1. Introduction

**Figure 1:**
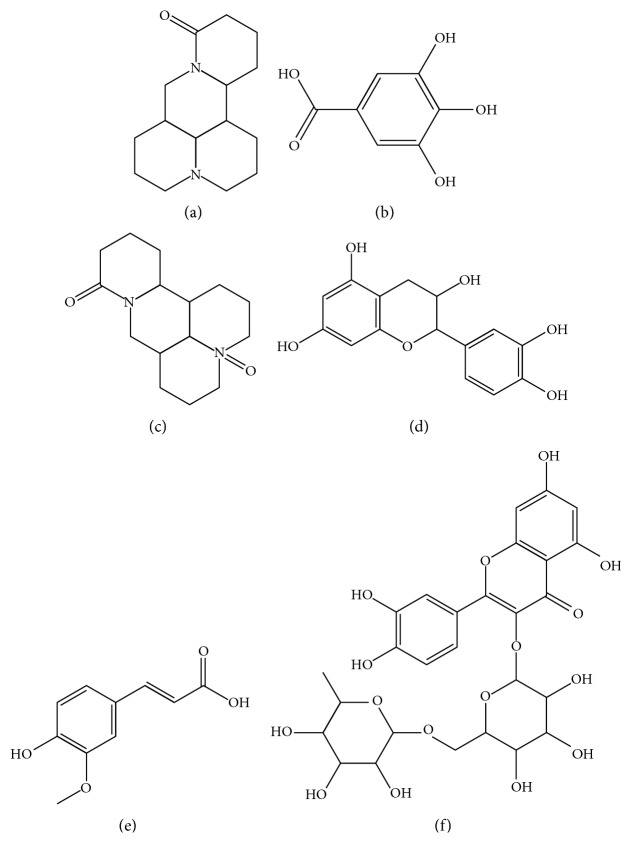
Chemical structure of six analytes: (a) matrine, (b) gallic acid, (c) oxymatrine, (d) catechin, (e) ferulic acid, and (f) rutin.

Danggui Kushen pills are a classical compound consisting of *Angelica sinensis* and *Sophora flavescens*, which have the effect of cooling blood and expelling dampness. It can be used for treatment of head and face sores, acne and pimples, eczema and itching, wine trough nose caused by blood dryness, and dampness heat [1–3]. In the “Ministry-Issued Traditional Chinese Materia Medica Preparation,” thin-layer chromatography (TLC) method was applied to identify the two herbs, *Angelica sinensis* and *Sophora flavescens*, in DKP. It was found that HPLC method was mainly used to control the quality of DKP through consulting literature. In addition, only ferulic acid, the active ingredient in *Angelica sinensis*, or matrine and oxymatrine, the main active component in *Sophora flavescens*, were determined [4–6]. However, other active ingredients with anti-inflammatory and antibacterial effects in this preparation, such as gallic acid, catechin, and rutin, are of equal importance in the treatment of acne and pimples [7–9]. Therefore, relying on single or several components to assess the quality of DKP is insufficient. It is extremely necessary to establish a low-cost, reliable, and efficient approach for quality evaluation of DKP. QAMS method has been considered as a good alternative for the quality control of traditional Chinese medicine and its preparation [10–12]. In this paper, a QAMS method for simultaneous determination of gallic acid, matrine, oxymatrine, catechin, ferulic acid, and rutin in DKP was established. Gallic acid was used as the internal reference standard due to its low price and easy acquisition. The proposed QAMS method laid foundation for the establishment of quality standard of DKP as well. The chemical structure of those six active components is shown in Figure 1.

## 2. Experimental

### 2.1. Instrument

Chromatographic separation was achieved on an Agilent 1260 high performance liquid chromatography (HPLC) system (including quaternary low pressure mixing pump, autosampler, column oven, diode array detector, and Chemstation workstation). An AB135-S electronic balance was obtained from Mettler Toledo International Co., Ltd. A DK-98-II type electric-heated thermostatic water bath pot was purchased from Tianjin Taisite Instrument Co., Ltd. A KQ-250 ultrasonic cleaner was purchased from Kunshan Ultrasonic Instrument Co., Ltd. An R series rotatory evaporator was acquired from Shanghai Shen Sheng Technology Co., Ltd.

### 2.2. Reagents and Materials

DKP was prepared by laboratory (batch number: 20171201, 20171202, 20171203, 20171204, and 20171205) according to the third volume of the “Ministry-Issued Traditional Chinese Materia Medica Preparation.” *Angelica sinensis* and *Sophora flavescens* were pulverized into powder, sieved, and mixed. Finally, 120–130 g of refined honey was added into 100 g mixed powder to obtain big honey pills: DKP.

Matrine (batch number: 110805-200508), gallic acid (batch number: 110831-201204), oxymatrine (batch number:110780-201007), catechins (batch number: 877-200001), ferulic acid (batch number: 110773-200611), and rutin (batch number: 100080-201409) were all purchased from China Pharmaceutical Biological Products Verification Institute (Beijing, China). Methanol (Fisher, America) was of chromatographic grade. Phosphoric acid and other reagents (Beijing Chemical Industry Factory) were all of analytical grade. Ultrapure water was purchased from Hangzhou Wahaha Co., Ltd.

### 2.3. Preparation of Mixed Standard Solution

Appropriate amount of matrine, gallic acid, oxymatrine, catechins, ferulic acid, and rutin was weighted precisely and dissolved with methanol to obtain stock standard solution with the concentration of 1191.8 *μ*g·mL^−1^, 11552.6 *μ*g·mL^−1^, 5.7 *μ*g·mL^−1^, 44.4 *μ*g·mL^−1^, 17.3 *μ*g·mL^−1^, and 848.2 *μ*g·mL^−1^, respectively. The standard solution above was stored at 4°C. Accurate 0.5 mL stock standard solution was placed into a 5 ml volumetric flask and dissolved with methanol (final adjusted volume 5 mL) to obtain the mixed standard solution with the concentration of matrine 119.18 *μ*g·mL^−1^, gallic acid 1155.26 *μ*g·mL^−1^, oxymatrine 0.57 *μ*g·mL^−1^, catechins 4.44 *μ*g·mL^−1^, ferulic acid 1.73 *μ*g·mL^−1^, and rutin 84.82 *μ*g·mL^−1^. The solution was shaken well and filtered through a 0.22 *μ*m membrane filter before HPLC analysis.

### 2.4. Preparation of Test Solution

About 5 g DKP was grinded and placed in a stoppered conical flask. Then 100 mL methanol was added into the conical flask and was ultrasonically extracted for 30 min. Ultrasonic extraction was repeated for three times. The extracts of each time were filtered and combined together. The combined filtrates were concentrated and evaporated to dryness via roller evaporator. Finally, the residue was redissolved with methanol (final adjusted volume 10 mL). The solution above was filtered through a 0.22 *μ*m microporous membrane filter after full shaking, and subsequent filtrate was collected as the test solution.

### 2.5. Preparation of Negative Control Solution

According to the prescription proportion, negative control samples without *Sophora flavescens* or *Angelica sinensis-Sophora flavescens* were taken and prepared into negative control solutions, respectively, based on the method in Section 2.4.

### 2.6. Chromatographic Conditions

Analysis was achieved on the Agilent ZORBAX SB-C18 column (250  mm × 4.6  mm, 5 *μ*m) along with the detective wavelength of 210 nm, 225 nm, 256 nm, and 320 nm. The mobile phase consisted of methanol (A) and 0.1% phosphoric acid solution (B). The flow rate was 1.0 mL·min^−1^, and the column temperature was maintained at 30°C. The injection volume of sample was 5 *μ*L. The concrete gradient elution conditions are displayed in Table 1.

### 2.7. Method Validation

#### 2.7.1. Specialization Test

**Figure 2:**
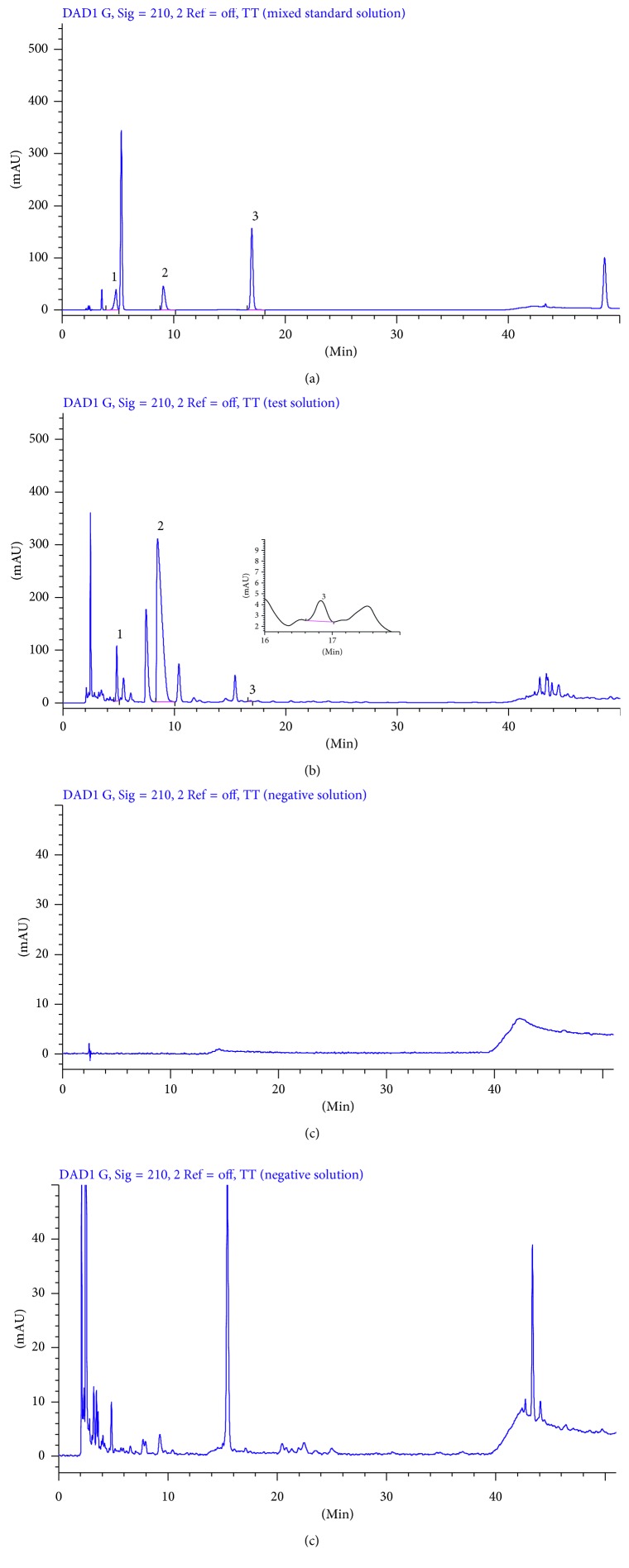
HPLC chromatograms of mixed standard solution (a), test solution (b), negative solution without *Angelica sinensis-Sophora flavescens*, (c) and negative solution without *Sophora flavescens* (d) at 210 nm (1: matrine; 2: oxymatrine; 3: catechin).

**Figure 3 fig3:**
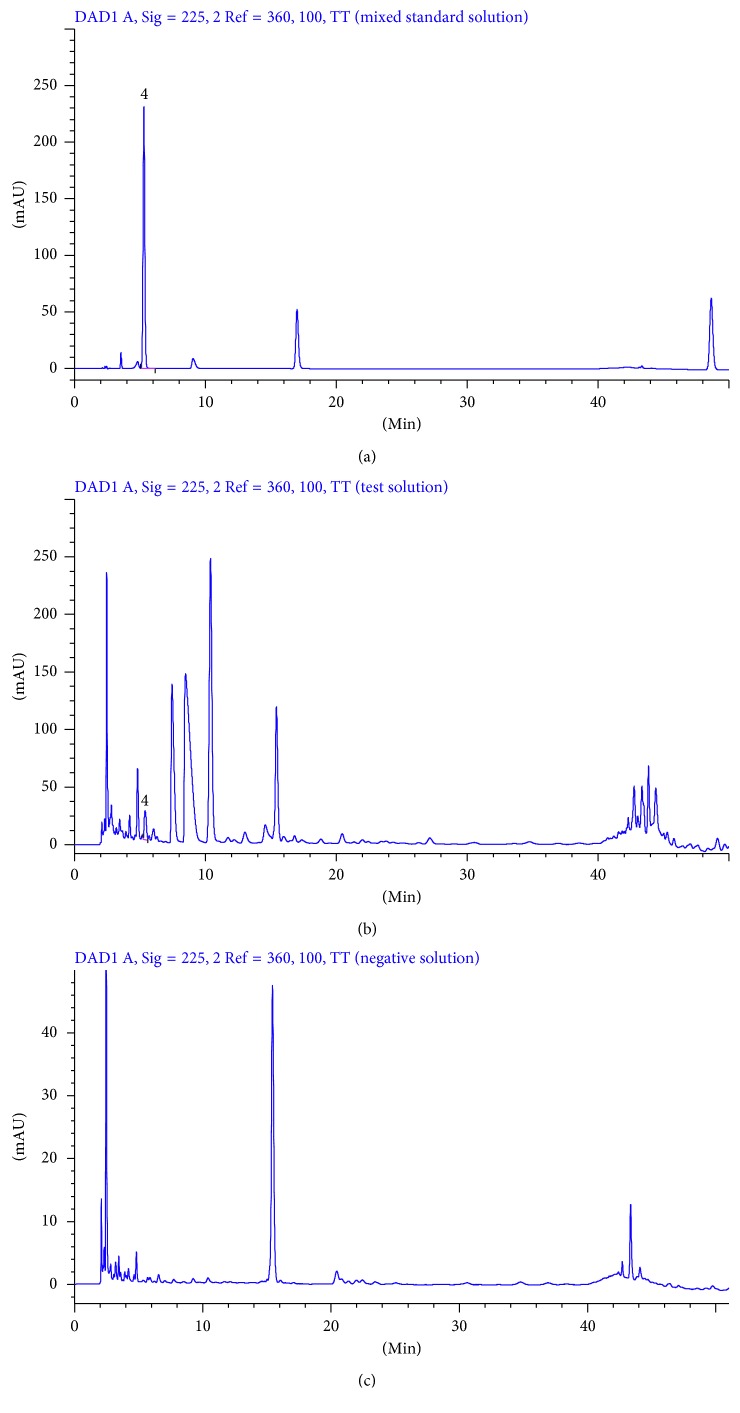
HPLC chromatograms of mixed standard solution (a), test solution (b), and negative solution without *Sophora flavescens* (c) at 225 nm (4: gallic acid).

**Figure 4 fig4:**
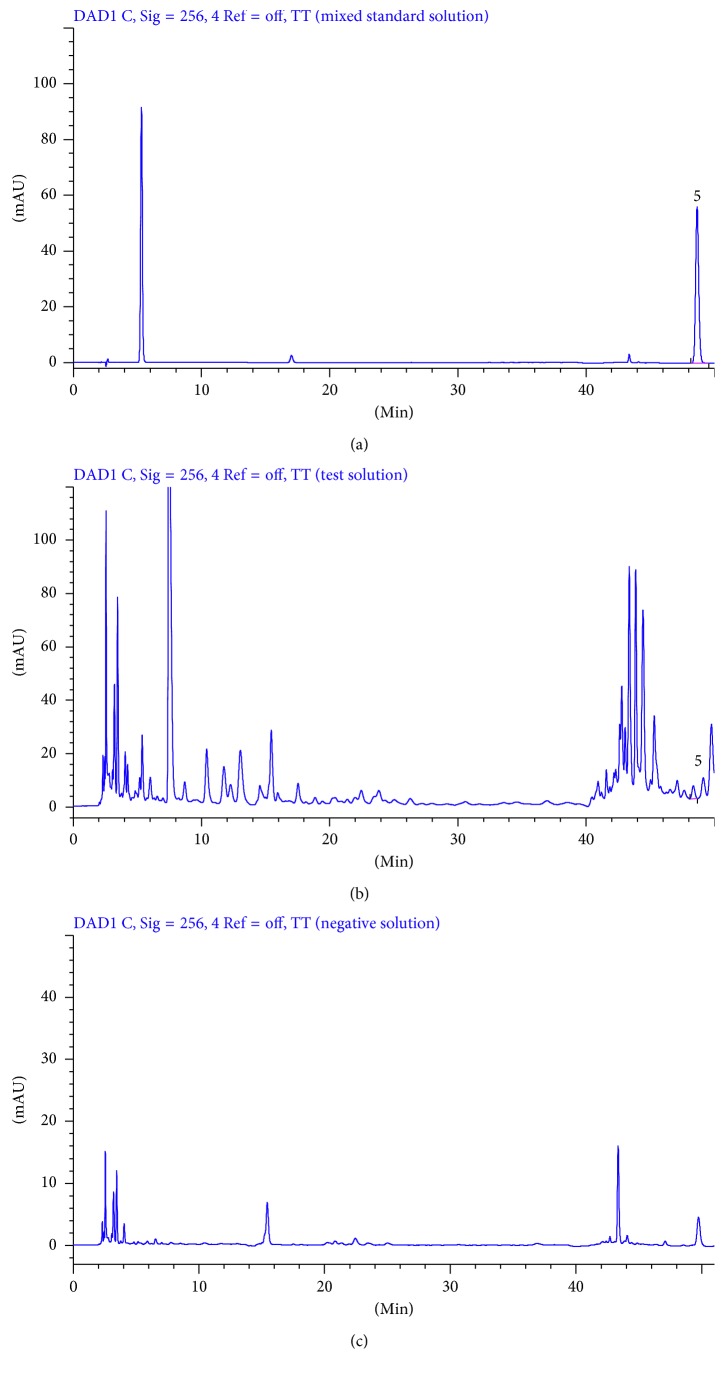
HPLC chromatograms of mixed standard solution (a), test solution (b), and negative solution without *Sophora flavescens* (c) at 256 nm (5: rutin).

**Figure 5:**
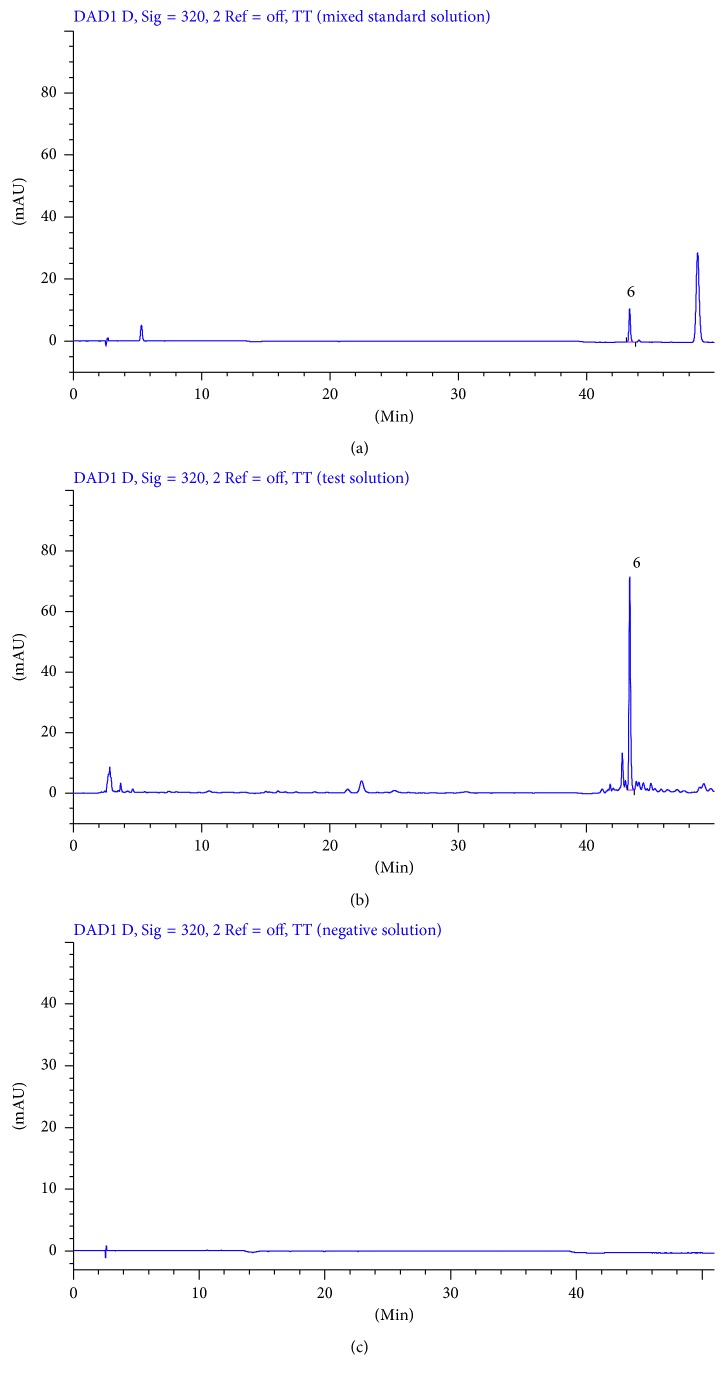
HPLC chromatograms of mixed standard solution (a), test solution (b), and negative solution without *Angelica sinensis-Sophora flavescens* (c) at 320 nm (6: ferulic acid).

Mixed standard solution, test solution, and negative control solution of each substance were injected into HPLC for analysis according to the chromatographic conditions under Section 2.6. The results are shown in Figures 2 [Fig fig3] [Fig fig4]–5.

#### 2.7.2. Linear Range

The stock standard solution (precise 0.1 mL, 0.25 mL, 0.5 mL, 1 mL, 2 mL, and 3 mL) was placed in a 5 ml volumetric flask and was dissolved with methanol (final adjusted volume 5 mL), respectively, to obtain the standard serial working solutions. Then the above working solutions were injected into the HPLC for analysis according to the chromatographic conditions under Section 2.6, respectively. In addition, the mixed standard solution was diluted with methanol. The limits of detection (LOD) and the limits of quantification (LOQ) were determined by three times and ten times of the signal noise ratio, respectively.

#### 2.7.3. Precision

An aliquot of mixed standard solution with the concentration of matrine 119.18 *μ*g·mL^−1^, gallic acid 1155.26 *μ*g·mL^−1^, oxymatrine 0.57 *μ*g·mL^−1^, catechins 4.44 *μ*g·mL^−1^, ferulic acid 1.73 *μ*g·mL^−1^, rutin 84.82 *μ*g·mL^−1^, and test solution prepared as Section 2.4 were taken and injected into HPLC for six continuous times according to the chromatographic conditions under Section 2.6, respectively. Besides, the chromatographic peak areas and RSDs of each components were recorded and calculated, respectively.

#### 2.7.4. Stability Test

An aliquot of same test solution (placed at room temperature) was injected into HPLC at 0, 2, 4, 8, 12, and 24 h for analysis according to the chromatographic conditions under Section 2.6, respectively. The peak area of each component was recorded, and RSD was calculated.

#### 2.7.5. Repeatability Test

Six aliquots of DKP with the same batch number were taken and prepared into six parallel test solutions following the method under Section 2.4 and injected into HPLC for analysis according to the chromatographic conditions under Section 2.6.

#### 2.7.6. Recovery Test

Six copies of 2.5 gram DKP(batch number: 20171201) with known content were accurately weighed and placed in stoppered conical flasks, respectively. Then 1 mL mixed standard solution (containing matrine 3120 *μ*g, gallic acid 30064 *μ*g, oxymatrine 7.5 *μ*g, catechins 105.4 *μ*g, rutin 40.3 *μ*g, and ferulic acid 1322.7 *μ*g per mL) was added into the conical flasks above and prepared respectively. The sample solutions were prepared according to the method under Section 2.4 and injected into HPLC for analysis following chromatographic conditions under Section 2.6, respectively. The peak areas were recorded, and average recovery rate and RSDs of each sample were calculated.

### 2.8. Establishment of QAMS Method

#### 2.8.1. Calculation of Relative Correction Factors (RCF)

3, 5, 10, 15, and 20 *μ*L of mixed standard solution prepared as Section 2.3 were taken and injected into HPLC for analysis according to the chromatographic conditions under Section 2.4, respectively. Besides, the chromatographic peak areas of each component were recorded.

#### 2.8.2. Reproducibility Test of RCF

Two different instruments (Agilent 1260 and Shimadzu LC-2030), three kinds of chromatographic columns (Agilent ZORBAX SB-C18, Agilent TC-C18, and Waters XBridge-C18 (250  mm × 4.6  mm, 5 *μ*m)), different flow rates (0.8, 1.0, and 1.2 mL·min^−1^), and different column temperatures (25, 30, and 35°C) were applied to investigate the influence of different conditions on RCF. The chromatographic peak areas of each component were recorded, and RCF of matrine, oxymatrine, catechin, ferulic acid, and rutin was calculated, respectively, using gallic acid as the internal reference.

### 2.9. Sample Content Determination

Test samples were prepared into test solutions according to the method under Section 2.4 and injected into HPLC for analysis following chromatographic conditions under Section 2.6, followed by calculation of contents of various constituents by external standard method as well as QAMS method.

## 3. Results and Discussion

### 3.1. Method Validation

#### 3.1.1. Specialization Test

The theoretical plates were selected according to the separation resolution between the analytes and impurities. After several tests, we found that the analytes have good resolution in chromatographic peaks when theoretical plates are bigger than or equal to 3000. As shown in Figures 2 [Fig fig3] [Fig fig4]–5, there was no interference in the corresponding position of the six components. In addition, the theoretical plate numbers of those constituents were not less than 3000. The separation degrees were all greater than 1.5, which indicated that the proposed method was of good specialization.

#### 3.1.2. Linear Range

The standard curve was drawn by using the chromatographic peak area (*Y*) as the vertical axis and the concentration of the reference solution (*X*) as abscissa. In addition, the limit of detection (S/N = 3) and the limit of quantification (S/N = 10) were calculated. The results are shown in Table 2, suggesting that six components presented good linear relationships in their determination ranges. LOD and LOQ of six substances were within the range of 0.01–0.11 *μ*g·mL^−1^ and 0.04–0.31 *μ*g·mL^−1^, which showed a high sensitivity under the established chromatographic condition.

#### 3.1.3. Precision

The RSD results of peak areas of matrine, gallic acid, oxymatrine, catechin, rutin, and ferulic acid in the test solution were 1.55%, 0.54%, 0.29%, 1.17%, 0.77%, and 0.94%, respectively. The RSD results of peak areas of matrine, gallic acid, oxymatrine, catechin, rutin, and ferulic acid in the mixed standard solution were 0.14%, 0.57%, 1.15%, 1.23%, 1.05%, and 0.22, respectively, which indicated that this method had good precision.

#### 3.1.4. Stability Test

The RSD results of area peaks of matrine, gallic acid, oxymatrine, catechin, rutin, and ferulic acid were 0.73%, 0.56%, 0.98%, 1.04%, 1.38%, and 0.88%, respectively, suggesting that test solution was stable within 24 h.

#### 3.1.5. Repeatability Test

The results showed that average mass fraction and RSDs of average mass fraction of matrine, gallic acid, oxymatrine, catechin, rutin, and ferulic acid were 0.1817%, 1.1255%, 0.0003%, 0.0048%, 0.0029%, and 0.0682% and 0.78%, 1.26%, 1.47%, 1.88%, 1.69%, and 1.82%, respectively, which indicated that this method was of good repeatability.

#### 3.1.6. Recovery Test

The content was determined and the recovery rate was calculated. The results showed that average recovery rate and RSD values of recovery rate of matrine, gallic acid, oxymatrine, catechin, rutin, and ferulic acid were 100.60%, 101.58%, 99.33%, 98.47%, 100.25%, and 101.04% and 1.37%, 1.69%, 1.64%, 1.78%, 1.87%, and 1.91%, respectively. The results illustrated that the proposed method was of good accuracy.

### 3.2. Establishment of QAMS Method

#### 3.2.1. Calculation of RCF

The RCF was calculated by multipoint correction method using the following calculation formula:

(1) $$\begin{array}{c} f_{s/k}=\frac{f_{s}}{f_{k}}=\frac{W_{k}\times A_{s}}{W_{s}\times A_{k}}, \end{array}$$

where *f*_*s*/*k*_ is the RCF of component to be measured, *A*_*s*_ is the peak area of internal reference, *W*_*s*_ is the concentration of internal reference, *A*_*k*_ is the peak area of component to be measured, and *W*_*k*_ is the concentration of component to be measured. Gallic acid was selected as the internal reference (1.000) for the quantitative analysis of other five components (matrine, oxymatrine, catechin, ferulic acid, and rutin) under the detection wavelengths of 210 nm, 225 nm, 256 nm, and 320 nm, respectively. The results are displayed in Table 3. It was found that the RCFs of matrine, oxymatrine, and catechin were 3.80, 3.03, and 0.39 under 210 nm. The RCF of rutin was 2.31 under 256 nm. The RCF of ferulic acid was 2.38 under 320 nm.

#### 3.2.2. Reproducibility Test of RCF

The results showed that RSD values of RCF measured in different conditions were all less than 2.27%, which indicated that the RCF calculated by the proposed method was of good reproducibility.

#### 3.2.3. Location of the Chromatographic Peak of Measured Component

The relative retention time was calculated according to the ratio of retention time of measured component (*k*) and internal reference (*s*). The calculation formula (2) is shown as follows:

(2) $$\begin{array}{c} Rt_{R}=\frac{t_{Rk}}{t_{Rs}}, \end{array}$$

where *Rt*_*R*_ is relative retention time, *t*_*Rk*_ is the retention time of component to be measured, and *t*_*Rs*_ is the retention time of internal reference. *Rt*_*R*_ between measured components and internal reference (gallic acid) was calculated via formula (2). In addition, RSDs of relative retention time measured in different conditions were calculated in order to evaluate the influence of different chromatographic systems, chromatographic column, column temperatures, and flow rates on *Rt*_*R*_. The results showed that RSDs of relative retention time between tested components and internal reference were all less than 1.65%, indicating that *Rt*_*R*_ was of good reproducibility and can be used to locate of the chromatographic peak of other five analytes. On the other hand, overall shape of chromatographic spectra of measured components can also be applied to the location of chromatographic peaks. In a word, chromatographic peaks can be accurately located in a great extent by the method established above.

#### 3.2.4. Comparison of the Results of QAMS Method and ES Method

The contents of each component in five batches of DKP were calculated by ES and QAMS method, respectively. The results are exhibited in Table 4. It was found that the relative errors (RE) of these two methods were less than 3.0%. There were no significant differences between the results of two determination methods, illustrating that the method established above was accurate and reliable.

### 3.3. Discussion

Traditional Chinese medicine compound preparations, as a traditional and classic medicine, have the characteristics of the diversity and complexity of traditional Chinese medicines as well. Therefore, the method for determining the content of traditional Chinese medicine compound preparations is gradually improving. The QAMS method has been widely used in traditional Chinese medicines and their decoction pieces and preparations [13–15]. Simultaneous determination of multiple components can be achieved via measurement of single component utilizing the function or proportion relationship between various components of traditional Chinese medicines. In this paper, QAMS method was applied to establish a new method for simultaneous determination of matrine, gallic acid, oxymatrine, catechin, ferulic acid, and rutin in DKP, which provided a better and convenient way to control the quality of DKP.

Gallic acid was selected as the internal reference of QAMS method in this study because gallic acid is one of the active ingredients of DKP and has pharmacological effects such as anti-inflammatory, antibacterial, antipathogenic microorganisms, antivirus, and so on. Gallic acid also has the advantage of low price and easy acquisition. Furthermore, there were no significant differences between the content determination results of QAMS method and ES method when gallic acid was used as internal reference. Taking the retention time and content of other components to be tested into consideration, gallic acid was considered as the internal reference finally.

The retention time of other measured components cannot be confirmed when QAMS method was applied because only one standard substance, internal reference (gallic acid), was used. Therefore, it was critical to locate the chromatographic peaks of components to be tested. In this study, retention time difference method and relative retention value method were investigated [16, 17]. The results showed that RSDs of retention time difference and relative retention value determined in different conditions were less than 6.06% and 1.65%, respectively. So, relative retention value method was used to locate the chromatographic peaks of components to be tested due to its good reproducibility and feasibility.

## 4. Conclusion

In this paper, the HPLC-DAD method was adopted for determination of relative retention time and relative correction factor of six active components in DKP for the first time. A QAMS method was developed for simultaneous determination of six active components in DKP. The QAMS method proposed in this study has been proved by methodology validation and found to be accurate, feasible, and reliable for contents determination of six active components in DKP, which can be applied to the quality control of DKP.

## Figures and Tables

**Table 1:** Gradient elution conditions.

Time (min)	*A* (%)	*B* (%)
0	13	87
10	13	87
11	19	81
36	19	81
39	40	60
53	40	60

**Table 2:** The linear equations, coefficient of determination, and linear range of six components.

Constituent	Linear equation	Linear range (*μ*g·mL^−1^)	*r*	LOD (*μ*g·mL^−1^)	LOQ (*μ*g·mL^−1^)
Matrine	*Y* = 6.9958*X* − 7.136	23.84∼715.08	0.9998	0.08	0.24
Gallic acid	*Y* = 27.728*X* − 3.6914	0.89∼26.64	0.9999	0.07	0.21
Oxymatrine	*Y* = 8.6405*X* + 138.3	231.05∼6931.56	0.9998	0.11	0.31
Catechin	*Y* = 37.684*X* + 4.6647	0.11∼3.42	0.9999	0.01	0.04
Rutin	*Y* = 10.578*X* + 1.9008	0.35∼10.38	0.9997	0.04	0.11
Ferulic acid	*Y* = 10.658*X* + 18.793	10.96∼328.92	0.9998	0.08	0.23

**Table 3:** Calculation results of RCFs.

Injection volume *μ*L	RCF				
	Matrine	Oxymatrine	Catechin	Rutin	Ferulic acid
3	3.80	2.99	0.38	2.29	2.37
5	3.85	3.11	0.39	2.31	2.36
10	3.81	3.03	0.39	2.30	2.41
15	3.73	2.99	0.39	2.30	2.37
20	3.82	3.01	0.38	2.33	2.40
Mean	3.80	3.03	0.39	2.31	2.38
RSD%	1.17	1.65	1.42	0.66	0.91

**Table 4:** Determination results and comparison of different methods.

Batch number	Method	Content (%)					
		Matrine	Gallic acid	Oxymatrine	Catechin	Rutin	Ferulic acid
20171201	ES	0.1190	0.0044	1.1552	3.1748*E* − 04	1.7485*E* − 03	0.0549
	QAMS	0.1170	0.0044	1.1697	3.0986*E* − 04	1.7752*E* − 03	0.0564
	RE%	1.68	—	−1.26	2.40	−1.53	−2.73

20171202	ES	0.1764	0.0061	1.3427	2.9770*E* − 04	2.2040*E* − 03	0.0713
	QAMS	0.1787	0.0061	1.3138	3.0130*E* − 04	2.1644*E* − 03	0.0719
	RE%	−1.30	—	2.15	−1.21	1.80	−0.84

20171203	ES	0.0985	0.0041	1.1542	3.5160*E* − 04	1.5428*E* − 03	0.0574
	QAMS	0.0993	0.0041	1.1347	3.4630*E* − 04	1.5763*E* − 03	0.0564
	RE%	−0.81	—	1.69	1.51	−2.17	1.74

20171204	ES	0.1242	0.0048	1.1787	3.0280*E* − 04	1.8037*E* − 03	0.0591
	QAMS	0.1228	0.0048	1.1567	2.9970*E* − 04	1.7855*E* − 03	0.0567
	RE%	1.13	—	1.87	1.02	1.01	4.06

20171205	ES	0.1130	0.0051	1.1679	3.4570*E* − 04	1.5994*E* − 03	0.0542
	QAMS	0.1106	0.0051	1.1462	3.4050*E* − 04	1.6311*E* − 03	0.0538
	RE%	2.12	—	1.86	1.50	−1.98	0.74

## Data Availability

The data used to support the findings of this study are included within the Supplementary Materials.

## Supplementary Materials

Supplementary Materials Table 1: the detailed results of recovery tests for six tested constituents of proposed QAMS method. Tables 2–4: the detailed results of reproducibility test of the relative correction factors (RCF), including effects of different instruments, chromatographic column, column temperature, and flow rate. Table 5: the effects of different instruments and chromatographic column on relative retention time (*tR*) and retention time difference (Δ*t*) of six tested components.
