# Supplementary material for: Simultaneous Determination of Six Active Components in Danggui Kushen Pills via Quantitative Analysis of Multicomponents by Single Marker
Source: J Anal Methods Chem. 2019 Jan 23;2019:9620571. doi: 10.1155/2019/9620571 (PMC6364111; doi:10.1155/2019/9620571)
Supplement: Supplementary Materials — Table 1: the detailed results of recovery tests for six tested constituents of proposed QAMS method. Tables 2–4: the detailed results of reproducibility test of the relative correction factors (RCF), including effects of different instruments, chromatographic column, column temperature, and flow rate. Table 5: the effects of different instruments and chromatographic column on relative retention time (tR) and retention time difference (Δt) of six tested components. [file 9620571.f1.pdf]

TABLE. 1 was the detailed results of recovery tests for six tested constituents of proposed QAMS method.

TABLE. 1 Results of recovery tests of various constituents(n=6)

Constituent	Known content /μg	Added amount/μg	Total measured amount/μg	Recovery rate(%)	Average recovery(%)	RSD (%)
matrine	2938.4	3120	6061.7	100.11		
	3131.9	3120	6248.4	99.89		
	3109.2	3120	6301.4	102.31	100.60	1.37
	2887.8	3120	6010.1	100.07		
	3201.6	3120	6392.4	102.27		
	3117.8	3120	6205.4	98.96		
oxymatrine	29281.7	30064	60152.7	102.68		
	28889.9	30064	58612.6	98.86		
	28673.4	30064	59318.3	101.93	101.58	1.69
	30258.1	30064	61494	103.90		
	28761.5	30064	59133.6	101.02		
	28652.8	30064	59034.7	101.06		
catechins	7.3	7.5	14.9	101.33		
	7.3	7.5	14.7	98.67		
	7.5	7.5	15.1	101.33	99.33	1.64
	7.4	7.5	14.8	98.67		
	7.4	7.5	14.7	97.33		
	7.5	7.5	14.9	98.67		
gallic acid	110.8	105.4	217.8	101.52		
	106.3	105.4	209.4	97.82		
	117.4	105.4	220.5	97.82	98.47	1.78
	113.7	105.4	215.7	96.77		
	109.5	105.4	214.4	99.53		
	106.6	105.4	209.2	97.34		
rutin	38.6	40.3	78.6	99.26		
	39.1	40.3	80.4	102.48		
	40.3	40.3	79.9	98.26	100.25	1.87
	40.1	40.3	80.2	99.50		
	39.7	40.3	81.1	102.73		
	40.7	40.3	80.7	99.26		
ferulic acid	1387.4	1322.7	2688.5	98.37		
	1402.7	1322.7	2774.9	103.74		
	1367.5	1322.7	2702.4	100.92	101.04	1.91
	1357.2	1322.7	2699.1	101.45		
	1428.5	1322.7	2781.5	102.29		
	1345.6	1322.7	2661.6	99.49		

TABLE. 2-4 displayed the detailed results of reproducibility test of the relative correction factors(RCF), including effects of different instrument, chromatographic column, column temperature and flow rate.

TABLE. 2 Effects of different instrument and chromatographic column on RCF

Instrument	Chromatographic column	Relative correction factors				
		Gallic acid/ matrine	Gallic acid / oxymatrine	Gallic acid / catechin	Gallic acid / rutin	Gallic acid / ferulic acid
Agilent 1260	Agilent ZORBAX SB-C_{18}	3.80	3.01	0.39	2.31	2.36
	Agilent TC-C_{18}	3.82	3.01	0.40	2.27	2.35
	Waters X Bridge-C_{18}	3.81	2.98	0.39	2.29	2.34
Shimadzu 2030	Agilent ZORBAX SB-C_{18}	3.85	2.99	0.40	2.31	2.38
	Agilent TC-C_{18}	3.79	3.07	0.38	2.35	2.27
	Waters X Bridge-C_{18}	3.73	3.03	0.39	2.25	2.33
Mean		3.80	3.02	0.39	2.30	2.34
RSD(%)		1.05	1.06	1.92	1.52	1.61

TABLE. 3 Effects of different column temperature on RCF

Column temperature	Relative correction factors				
	Gallic acid/ matrine	Gallic acid / oxymatrine	Gallic acid / catechin	Gallic acid / rutin	Gallic acid / ferulic acid
25℃	3.78	3.03	0.39	2.33	2.40
30℃	3.81	2.96	0.39	2.32	2.37
35℃	3.65	3.01	0.38	2.28	2.42
Mean	3.75	3.00	0.39	2.31	2.40
RSD(%)	2.27	1.20	1.49	1.15	1.05

TABLE. 4 Effects of different flow rate on RCF

flow rate (mL • min^{-1})	relative correction factors				
	Gallic acid/ matrine	Gallic acid / oxymatrine	Gallic acid / catechin	Gallic acid / rutin	Gallic acid / ferulic acid
0.8	3.82	3.07	0.40	2.31	2.38
1.0	3.79	2.99	0.41	2.34	2.41
1.2	3.73	3.02	0.39	2.27	2.33
Mean	3.78	3.03	0.40	2.31	2.37
RSD(%)	1.21	1.34	2.50	1.52	1.70

TABLE. 5 showed the effects of different instrument and chromatographic column on relative retention time(t_R) and retention time difference(Δt) of six tested components.

TABLE. 5 Results of t_R and Δt of tested components

Instrument	Column	Matrine		Oxymatrine		Catechin		Rutin		Ferulic acid	
		t_R	Δt	t_R	Δt	t_R	Δt	t_R	Δt	t_R	Δt
Agilent 1260	Agilent ZORBAX SB-C_{18}	0.91	0.479	1.707	3.760	3.200	11.702	9.198	42.344	8.154	38.460
	Agilent TC-C_{18}	0.895	0.531	1.671	3.937	3.085	11.873	9.224	44.594	8.009	36.794
	Waters X Bridge-C_{18}	0.897	0.542	1.702	3.885	3.190	11.795	9.118	43.658	8.145	38.880
Shimadu 2030	Agilent ZORBAX SB-C_{18}	0.913	0.484	1.702	3.781	3.202	12.064	9.153	42.417	8.155	38.481
	Agilent TC-C_{18}	0.892	0.538	1.675	3.952	3.107	12.271	9.211	44.672	8.109	36.802
	Waters X Bridge-C_{18}	0.881	0.553	1.692	3.879	3.191	12.316	9.12	43.701	8.145	38.847
	Mean	0.898	0.521	1.692	3.866	3.163	12.004	9.171	43.564	8.120	38.044
	RSD(%)	1.32	6.06	0.90	2.05	1.65	2.12	0.51	2.32	0.70	2.58
